# Understanding the long-term clinical effectiveness of L’Episcopo procedure in restoring external rotation & abduction in patients suffering from brachial plexus birth injury: a systematic review & meta-analysis

**DOI:** 10.1007/s00402-026-06191-w

**Published:** 2026-02-02

**Authors:** Kunal P Shah, Jonathan Elias, Ryan St. John, Mario Salah, Kunal Damaraju, Sean McMillan

**Affiliations:** 1 Rowan University School of Osteopathic Medicine, Rowan University, Glassboro, USA; 2 Futures Forward Research Institute, Toms River, USA; 3 Department of Orthopedics, Virtua Health, Marlton, USA; 4 Inspira Medical Center Vineland, Vineland, USA

**Keywords:** Brachial Plexus; Brachial Plexus Birth Injury; BPBI; Tendon Transfer; L’episcopo; L'Episcopo' Latissimus Dorsi transfer; Tere Major transfer

## Abstract

**Introduction:**

Brachial Plexus Birth Injury (BPBI) affects 1 in 1,000 births in the U.S., drawing significant attention from orthopedic and plastic surgeons. While risk factors include shoulder dystocia, instrumental delivery, and fetal macrosomia, many cases lack clear causes. Tendon transfers, especially L’Episcopo, are commonly performed to restore shoulder external rotation and abduction using latissimus dorsi (LD) and teres major (TM) transfers.

**Purpose:**

The goal is to understand the clinical effectiveness of L’Episcopo in improving shoulder ROM and complications in BPBI patients.

**Methods:**

A comprehensive search was conducted using PRISMA 2020 guidelines, yielding 612 studies. After the initial inclusion and exclusion, 10 studies were reviewed in full text using Rayyan.ai. Studies for patients undergoing the L’Episcopo procedure for BPBI, with Mallet scores assessing shoulder range of motion, were included as inclusion criteria. Statistical analysis was performed for a random-effects meta-analysis using SPSS.

**Results:**

Four studies were included in the meta-analysis. Mallet scores were assessed at an average follow-up of 44 months. L’Episcopo procedure demonstrated both clinical and statistical effectiveness, with a Hedge’s g value of 1.66 indicating a very large effect size, and a p-value of < 0.0001. An improvement in Mallet scores is associated with increased range of motion and functionality of the shoulder.

**Conclusions:**

L’Episcopo with the use of Latissimus Dorsi and Teres major shows supportive evidence in improving external rotation and abduction in patients suffering from brachial plexus birth injury (BPBI).

## Introduction

 Brachial plexus birth injuries (BPBI) affect nerve fibers that make up the brachial plexus, composed of the C5-T1 anterior rami, and occur in approximately 0.5 to 3 per 1000 live births [1, 2]. Non-operative treatment and spontaneous recovery are possible, but many require surgical intervention. Of those who do require surgical intervention, there are still 1 in 5 who retain some degree of permanent disability [3]. BPBI risk increases with various factors such as vacuum or forceps-assisted delivery, fetal macrosomia, gestational diabetes, and shoulder dystocia [4, 5]. The resulting functional limitations significantly impact activities of daily living and long-term quality of life.

Despite advances in diagnosis and surgical technique, outcomes remain variable due to ongoing debate over optimal classification systems, diagnostic methods, and treatment approaches [6]. BPBI is commonly differentiated between upper plexus injuries (C5, C6, ± C7; Duchenne-Erb) and lower motor plexus injuries (C8-T1; Klumpke), with the former predominating and leading to the greatest disability [7]. Treatments aim to restore hand function, elbow flexion, and shoulder abduction and external rotation through approaches such as neurolysis, neuroma resection and grafting, primary nerve transfers, and tendinous transfers such as the L’Episcopo procedure [8–11]. However, no consensus exists regarding the most effective long-term intervention.

Joseph B. first described L’Episcopoin in 1934 using an anterior and posterior approach to transfer the latissimus dorsi and teres major laterally to restore external rotation [12]. Subsequently, Hoffer et al. modified this procedure to have the tendinous insertions on the posterior and superior rotator cuff to address Erb’s palsy [13]. Both variations have demonstrated improvements in shoulder function, yet the durability of these benefits remains unclear. Previous reviews comparing tendon transfers and nerve grafts suggest higher short-term success with tendon transfers. A theory to explain this is that tendon transfers utilize already functional tendons to restore lost function, while nerve grafts have to make new axonal connections to denervated tissues [14]. The long-term durability of the L’Episcopo procedure has not been systematically evaluated, representing a critical gap in guiding surgical decision-making. This systematic review and meta-analysis aims to address the current lack of pooled long-term functional outcomes of those who underwent the original L’Episcopo procedure to treat BPBI to better guide clinical judgment and inform clinicians of this procedure’s utility.

## Methods

A systematic review and meta-analysis were conducted following the 2020 Preferred Reporting Items for Systematic Reviews and Meta-Analyses (PRISMA) guidelines [15].

### Search procedure

A comprehensive review of five major scientific databases was conducted to evaluate the effectiveness of the L’Episcopo procedure for BPBI. On February 6th, 2025, PubMed, Embase, Scopus, Web of Science, and Cochrane Library were searched utilizing the following string: (Latissimus Dorsi Tendon Transfer) OR (Teres Major Tendon Transfer) OR (L’Episcopo) OR (LEpiscopo) AND (“Brachial Plexus Neuropathy” OR “Brachial Plexopathy” OR “Brachial Plexus Disease” OR “Brachial Plexus” OR “Brachial Plexus Birth Injury” OR “Erb Paralysis” OR “Erb’s Palsy” OR “Erb Palsy” OR “Erbs Palsy” OR “Klumpke Paralysis” OR “Klumpke’s Palsy” OR “Klumpke Palsy” OR “Klumpkes Palsy” OR “Palsy, Klumpke’s” OR “Lower Brachial Plexus Palsy” OR “Lower Brachial Plexus Neuropathy” OR “Middle Brachial Plexus Neuropathy” OR “Upper Brachial Plexus Neuropathy”). Key terms were identified using the Medical Subject Headings (MeSH) tool.

### Inclusion and exclusion criteria

Articles included in this meta-analysis were retrospective and prospective studies. Studies that used the L’episcopo procedure for the management of BPBI were conducted on patients under 18 years old, and included pre- and postoperative modified Mallet scores presented in mean and standard deviation (SD). No restrictions were placed on the study date range for inclusion. Articles excluded were cross-sectional studies, case studies, and case series, since they provide anecdotal evidence with very small sample sizes, high susceptibility to reporting bias, and heterogeneous outcome measures. Given the focus of this review on long-term surgical outcomes, only cohort studies and higher-quality designs were included to enhance the validity and generalizability of results. Articles that did not use the L’Episcopo procedure for BPBI on patients under the age of 18 years, or did not report the pre- and postoperative modified Mallet score mean and SD, were excluded from this review.

### Study selection

**Fig. 1:**
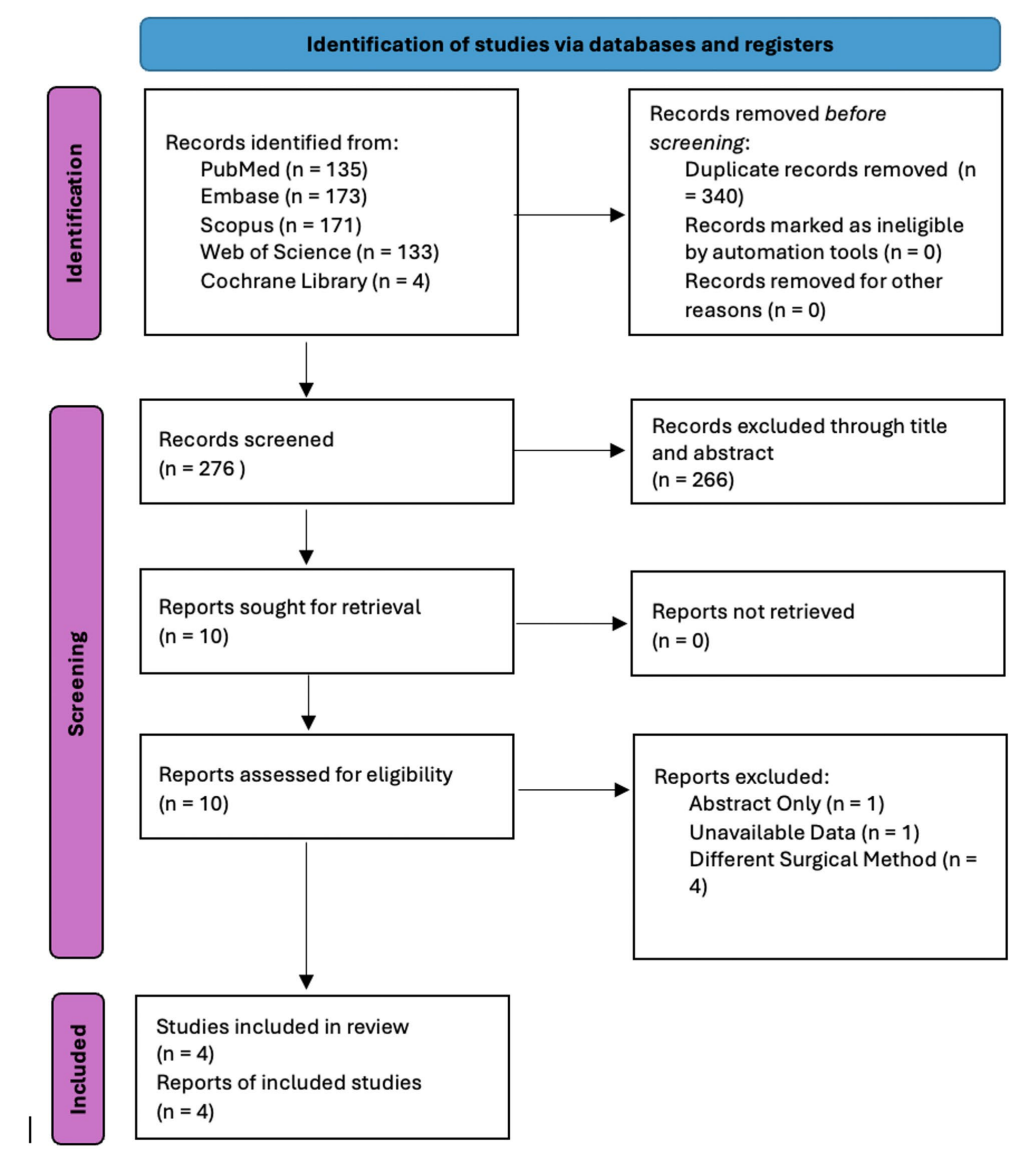
PRISMA diagram portraying the selection process

A total of 616 studies were retrieved using our search string. The retrieved studies were imported into Rayyan.ai for screening. Artificial intelligence was used to detect duplicates; however, its findings were confirmed by two authors (KS and JE) before deletion. Any disagreements between the two authors were resolved by a third author (RSJ). Following the deletion of duplicate articles, the abstracts and titles of the remaining 276 studies were screened, excluding 266 studies in the process. 10 studies were sought for retrieval and effectively retrieved. The reviewers thoroughly screened the full-length articles, eliminating 6 studies that did not fit our search criteria. Figure 1 portrays the PRISMA flow diagram regarding the selection process.

### Data extraction

Qualitative and quantitative data were gathered from the included studies after the article selection process. All data was collected independently by four reviewers, with any conflicts resolved by a fifth author (KD). The pre- and postoperative modified Mallet scores were extracted onto a Microsoft Excel sheet in mean and SD format. Follow-up time, pre- and postoperative number of participants, outcomes, year of publication, and study authors were also collected. Mallet scores were used as the primary outcomes for our study. If any included retrospective or prospective study contained multiple groups, only the data regarding the L’Episcopo procedure were collected and included in our analysis.

### Outcomes measured

The modified Mallet score is a clinical assessment tool to evaluate active shoulder functions for infants and children who have sustained BPBI. The score is determined by evaluating abduction, external rotation, hand to back of neck, hand to lower spine, and hand to mouth movements. Each action is graded from 0 to 5 (5 = normal and symmetrical movement, 0 meaning no movement) and summed up for a modified Mallet score [16].

### Statistical analysis

Statistical analysis was performed using the RStudio package version 4.5.1. The meta-analysis utilizing a random effects model aggregated the effect sizes of all included studies, allowing for the evaluation of the mean Mallet score in the postoperative group compared with the mean Mallet score in the preoperative group. Improvements in the postoperative outcomes were determined by the effect size of the analysis (Hedge’s g) with 95% confidence intervals. A Hedge’s g value of > 0.8 indicates a large clinical significance [17]. A test of between-subgroup homogeneity with a p value < 0.05 was used to determine statistical significance between the pre-operative and post-operative groups [18].

Heterogeneity was determined by I^2^ ratio (I^2^ = τ^2^ /H^2^). Larger I^2^ values suggest variance between the studies. Tau-squared (τ^2^) represents the variation in between effect sizes beyond random chance. A random-effects model was employed to assess expected variance not due to an external variable. The heterogeneity p-value was also used to determine the significance of the overall heterogeneity.

### Sensitivity analysis

A sensitivity analysis was conducted following the assessment of heterogeneity or publication bias using a funnel plot. To evaluate the robustness of the data, the results will be re-analyzed after removing the outliers. The re-analysis will be performed using a random effects model for Hedge’s g in RStudio to overcome any potential outliers that could impact the results of the measured outcomes.

### Risk of bias and certainty of evidence assessment

The included studies’ methodological quality was evaluated using the modified Grading of Recommendations Assessment, Development and Evaluation (GRADE) criteria. Bias in the included studies was assessed independently by two reviewers. Since our study included different levels of clinical studies, ROBINS-I was used to evaluate their bias.

## Results

### Summary of included studies

Within this systematic review and meta-analysis, four retrospective and prospective studies that involved using L’Episcopo as management for BPBI with the required inclusion criteria were included [19–22].

Topley et al. conducted a retrospective study that compared children with a mean age of 6 years and BPBI’s who had undergone a single tendon transfer from the teres major (*N* = 13) or a double tendon transfer from both the teres major and latissimus dorsi (*N* = 13). Three patients were recruited prospectively to fulfill matching requirements. The outcomes assessed after a mean follow-up time of 48 months were modified Mallet scores in various planes of motion of the arm between pre- and post-op, along with joint angles of the glenohumeral and humerothoracic joints. The results showed that internal rotation had a greater motion with the double tendon transfer group at the glenohumeral and humerothoracic joints. Importantly, this study was only able to assess 11 patients’ pre-operative Mallet scores, as opposed to the 13 patients’ postoperative Mallet scores, which the author attributes to the patients’ inability to participate in the examination due to their young age.

De Luna et al. conducted a prospective study of patients (*N* = 16) with an average age of 10 years and 7 months who underwent a Sever-L’Episcopo procedure. The mean follow-up time was 58 months and 15 days. The outcome measures included Mallet scores and correlational differences with age and the lengths of follow-ups. The study found a significant improvement in Mallet scores between pre- and post-op measurements, showing that the procedure improved upper limb function.

Waters and Bae conducted a prospective study that analyzed patients with BPBP (*N* = 25), and a mean age of 42 months, who underwent teres major and Latissimus dorsi tendon transfer and soft-tissue lengthening at pre-op and a minimum of 2-year follow-up, with a mean follow-up time of 43 months. The results showed that all patients had a clinical improvement in all elements of the modified Mallet scores, including global abduction, global external rotation, hand-to-neck motion, hand-to-spine motion, and hand-to-mouth motion, with a statistical significance of *p* < 0.01. Additionally, glenoid retroversion and humeral head subluxation were analyzed, where the results showed changes in the mean orientation of both measurements.

Berkoz et al. conducted a retrospective study in 2023 that also looked at 14 patients, with a mean age of 8 years, who had undergone a latissimus dorsi and teres major tendon transfer in cases of BPBP, along with either two rounds of a botulinum toxin injection (*N* = 7) or no injection (*N* = 7). After a mean follow-up period of 27 months, the outcome measures included mean latissimus dorsi myocyte thickness, Mallet scores, and shoulder abduction, flexion, external, and internal rotation. The results showed a non-significant increase in both groups for the modified Mallet scores. Further summary of all the included studies can be found below in Table 1.

**Table 1:** Summary of the included studies

Author	Year	*N*	Design	Mean follow-up	Mean age	Intervention	Outcome
Topley et al.	2022	*N* = 13 (teres major single tendon transfer) and 13 (latissimus dorsi and teres major double tendon transfer)	Retrospective	48 months	6 years	Single-tendon transfer with the teres major vs. Double-tendon transfer with latissimus dorsi and teres major	Mallet scores, joint angles of the glenohumeral and humerothoracic joints
de Luna et al.	2012	*N* = 16	Prospective	58 months + 15 days	10 years and 7 months	Underwent a Sever-L’Episcopo procedure	Mallet scores and correlational differences with age and length of follow-up
Waters et al.	2005	*N* = 25	Prospective	43 months	42 months	Teres major and Latissimus dorsi tendon transfer with soft-tissue lengthening	Mallet scores, glenoid retroversion, humeral head subluxation
Berkoz et al.	2023	*N* = 7 (Botox injection) and 7 (no injection)	Retrospective	27 months	8 years	Teres major and Latissimus dorsi tendon transfer with either 2 rounds of Botox injection or no injection	Mallet scores, latissimus dorsi myocyte thickness, shoulder abduction/flexion/external rotation/internal rotation

### Group demographics

Four studies were included in this systematic review and meta-analysis, yielding an evaluation of 59 distinct patients. The mean age at the time of operation of the included sample was 6.2 years, with a range of 6 months to 10 years.

### Effects of intervention

**Fig. 2:**
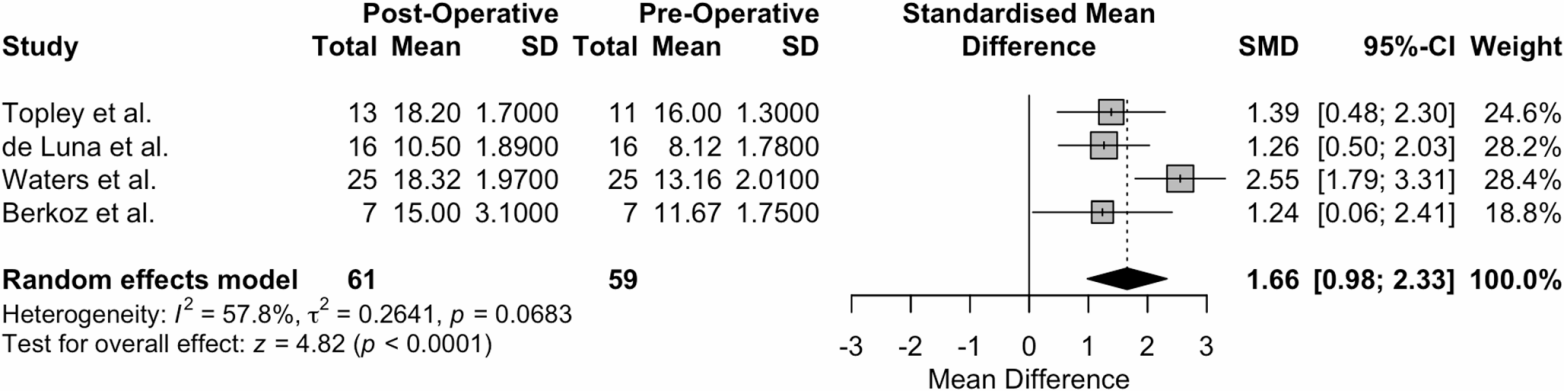
Forest plot of the Mallet scores of the four included studies, with a mean follow-up of 46.24 months

The mean and SD of the pre-operative and post-operative Mallet scores are portrayed in Table 2. Regarding the postoperative compared to the preoperative Mallet score, a test of effect size using a random effects model with a mean follow-up of 46.24 months, with a range of 27 months to 58 months, revealed clinically and statistically significant improvement across the four studies (Hedge’s g = 1.66, *p* < 0.0001, 95% [0.98, 2.33]). The results of the meta-analysis are portrayed in Fig. 2 in forest plot format.

**Table 2:** Pre-operative and post-operative mallet scores presented as means ± SD

Variable	Pre-operative (*n* = 59)	Post-operative (*n* = 61)
Mallet Score	12.15 ± 3.3	15.9 ± 3.93

**Fig. 3:**
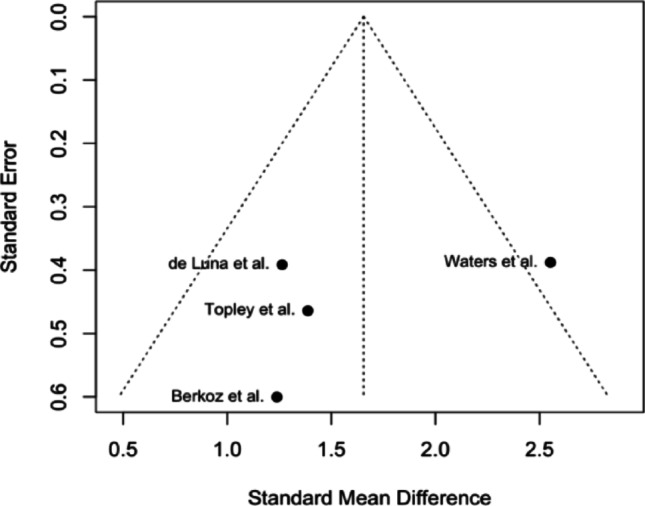
Funnel plot of the included studies

A funnel plot was generated using the included studies. The plot demonstrates asymmetry, with a cluster of studies yielding comparable effect sizes, while the study conducted by Waters et al. is positioned far to the right outside the pseudo 95% confidence interval of the pooled estimate, indicating a possibility for high heterogeneity. The results are portrayed in Fig. 3.

### Risk of Bias and Certainty Assessment

The GRADE analysis for the certainty of the evidence was performed by two independent authors and yielded three “low” grade studies and one “moderate” grade study. Primarily, the studies initially received a “low” grade due to being either retrospective or non-randomized [23]. However, the study conducted by Waters and Bae was upgraded to a “moderate” grade due to portraying significant magnitudes of the effect of the intervention [24]. Additionally, all included studies portrayed “not serious” levels of risk throughout the five domains [24]. An overall GRADE analysis is presented in Table 3.

**Table 3:** A GRADE analysis of the four included studies, assessing their level of evidence

Author	Year	Study design	Risk of bias	Inconsistency	Indirectness	Imprecision	Publication bias	Grade
Topley	2022	Retrospective	Not serious	Not serious	Not serious	Not serious	Not serious	Low
De Luna	2012	Prospective	Not serious	Not serious	Not serious	Not serious	Not serious	Low
Waters	2005	Prospective	Not serious	Not serious	Not serious	Not serious	Not serious	Moderate
Berkoz	2023	Retrospective	Not serious	Not serious	Not serious	Not serious	Not serious	Low

**Fig. 4:**
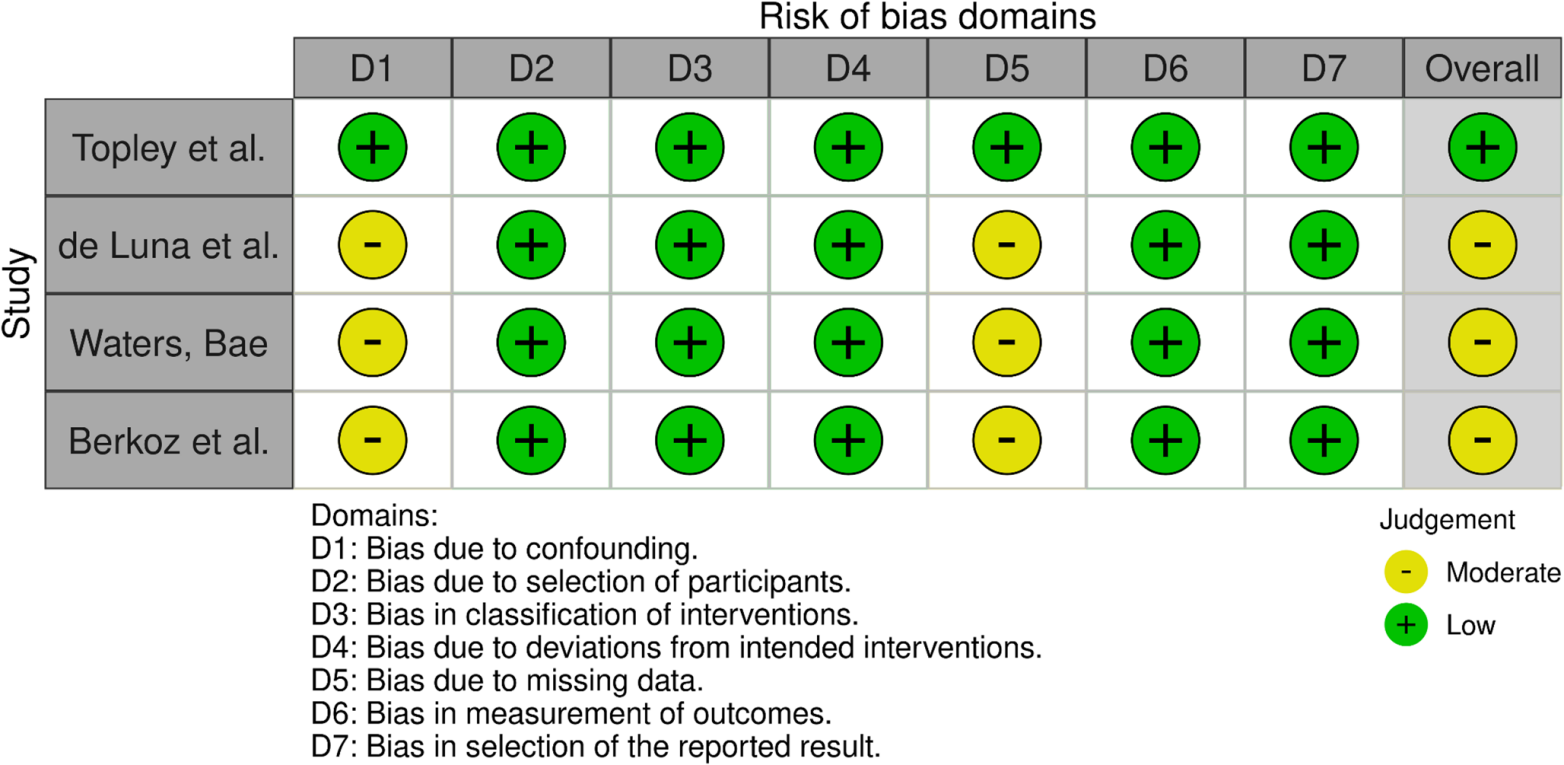
Traffic light plot portraying the risk of bias for each domain in ROBINS-1 regarding the included studies

**Fig. 5:**
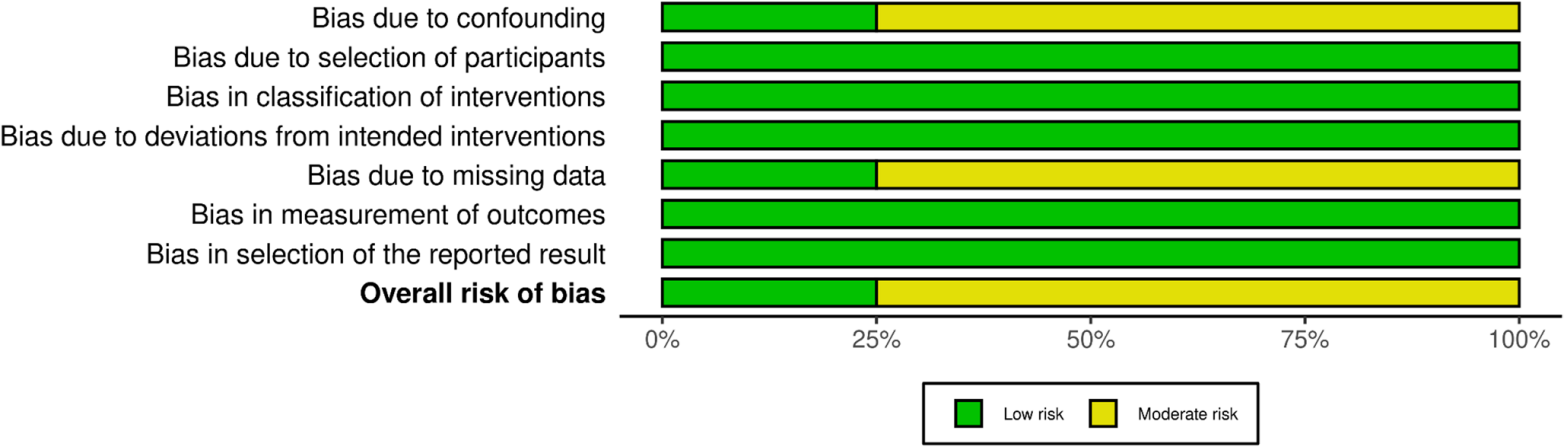
Summary plot of the ROBINS-I assessment for the included studies

A risk of bias assessment for the included studies was performed based on the Cochrane Risk of Bias in Non-randomized Studies-I (ROBINS-I) tool by the same two authors. A ROBINS-I analysis was performed since the studies included were not RCTs. Three studies were of moderate risk of bias, while one study was assessed to be of low risk of bias. Regarding the moderate-level studies, D1 and D5 were the domains in which the articles had an increased risk of bias. Each domain for the ROBINS-I evaluation is represented in Figs. 4 and 5 via the ROBVIS tool [25].

### Sensitivity analysis

**Fig. 6:**
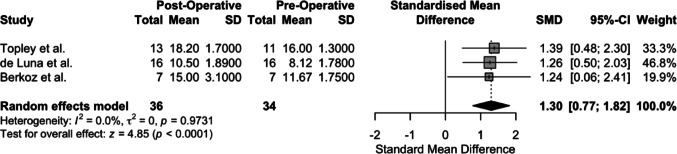
Forest plot analysis after sensitivity analysis by eliminating the Waters et al. study

**Fig. 7:**
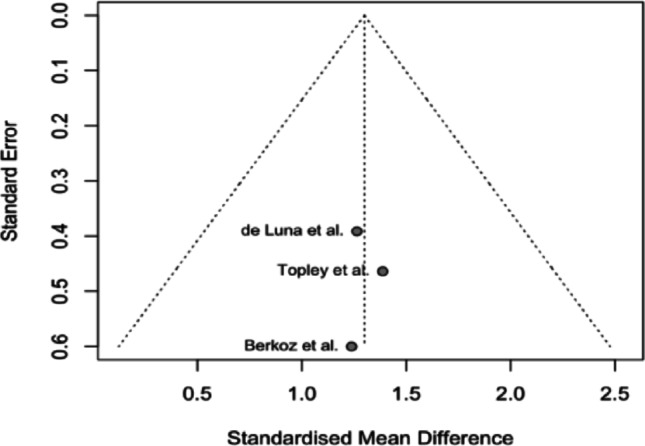
Funnel plot of the included studies post-sensitivity analysis

Based on the funnel plot analysis, a sensitivity assessment was performed by eliminating the Waters et al. study; heterogeneity of the included studies analysis dropped to zero (τ^2^ = 0.000, I^2^ = 0.0%, *p* = 0.9731), indicating the source of heterogeneity due to possible variations in patient population or bias contributing to the analysis. Hedge’s g values from the random effects analysis were statistically and clinically significant (Hedge’s g = 1.30, *p* < 0.0001) (Figs. 6 and 7).

## Discussion

The results of this systematic review and meta-analysis demonstrate L’Episcopo as an effective and durable improvement in shoulder function for children with BPBI, with a large effect size (Hedge’s g = 1.66, *p* < 0.0001) sustained over an average follow-up of 44 months. An increase in Mallet scores reflects clinically significant recovery of external rotation, abduction, and functional reach, highlighting the procedure’s ability to restore activities of daily living. Sensitivity analysis identified age-related variation as the main driver of heterogeneity, with younger patients showing greater variability in outcomes. Despite differences in study design, the consistency of benefit across included cohorts reinforces L’Episcopo as an effective reconstructive option for long-term shoulder function.

Current literature revolving around the use of L’Episcopo as an intervention supports these findings. Mehlman et al. 2011 study provided an in-depth analysis of tendon transfers associated with L’Episcopo leading to significant improvement in global shoulder motions, with little to no loss of internal rotation [26]. A recent literature review performed by Brogan in 2019 critically evaluates the use of L’Episcopo as an effective intervention for patients of BPBI. While complications exist around the maneuverability of the shoulder motion during the extraction and transfer, particularly revolving around brachial plexus neurapraxia from excessive anterior retraction during exposure, axillary or radial nerve injury, or posterior brachial plexus damage due to blind, imprecise dissection of LD/TM insertion, thoracodorsal nerve injury from over-mobilization of LD muscle or tendon [11].

While the use of L’Episcopo is not a novel innovation, newer variations of the procedure have been developed. Once such a modification involves the subscapularis tendon through lengthening or release of the muscle. While the newer technique seems promising in improving the external rotation and abduction of the shoulder, major concerns arise regarding the contractures of internal rotation (IR) as the subscapularis is primarily involved in IR and compromises anterior stabilization. The sliding technique of the subscapularis aims to provide better clinical outcomes in preservation of IR and stability compared to release [27]. These are double-muscle tendon transfers, but recent literature and clinical intervention share a possibility in the use of single-muscle tendon transfers, like utilizing the LD only, TM only, or even the pectoralis major [28–32]. While the single muscle transfers preserve the second muscle transfer, ultimately reducing donor site morbidity, less procedural time and associated complications, quicker recovery, and potential choice of second graft if revision surgery is required. Studies comparing single muscle tendon transfers, such as Ibrahim et al. 2023 show that the TM has proven more effective with a minor loss of internal rotations compared to LD [30]. With LD transfers, there were high failure rates in patients with upper trunk injuries needing a subsequent TM transfer as well in the future [33, 34]. With Topley et al. 2022 comparing TM only to L’Episcopo, double muscle tendon transfer provides higher internal rotation preservation with no difference in restoration of external rotation and abduction [19].

This recent shift in the differing clinical approaches demonstrates a need for clear head-to-head randomized controlled trials utilizing standard outcomes of diagnosis, like the mallet score and evaluations, in understanding these strategies in improving global shoulder range of motion in BPBI. As with the current landscape, there is a lack of research comparing which techniques aim to provide better outcomes in the management of BPBI. There is no clear evidence which technique provides significantly better outcomes, but L-Episcopo has been used as the gold standard until now; these newer strategies are not conclusive enough to change the standard of care. Modifications show improvement on an individual basis, but a lack of randomized trials directly comparing these techniques is a potential hindrance for researchers and orthopedic surgeons to better understand and help patients.

### Limitations

Our study is not without limitations, with a very limited sample patient population of 59, and an analysis of prospective and retrospective studies, there stems a moderate amount of heterogeneity in the analysis (τ^2^ = 0.2641, I^2^ = 57.8%, *p* = 0.0683). This heterogeneity may be a result of the inclusion of non-head-to-head studies as part of our data collection and analysis, wherein if a study of interest was comparing L’Episcopo to another surgical approach in the management of BPBI, only the data regarding L’Episcopo were collected and analyzed. The reason for approaching this route was due to the lack of evidence available to perform a clear head-to-head analysis comparing various surgical interventions. Additionally, the relatively small sample patient population reduces the power of our review and may lead to less generalizable findings. With the high initial heterogeneity, a sensitivity analysis was conducted. In the sensitivity analysis, the study conducted by Waters et al. was excluded as it portrayed significant asymmetry from the other studies in the funnel plot, seen in Fig. 3. Excluding Waters et al. yielded (τ^2^ = 0.000, I ^2^ = 0.0%, *p* = 0.9731), showing no heterogeneity without this outlier, potentially outlining underlying bias or variation in the Waters et al. study compared to other included studies. One possible explanation as to why the study conducted by Waters et al. was an outlier in our analysis may be the younger age of patients. The mean age of that study was approximately 3.5 years, while Topley, de Luna, and Berkoz had approximate mean ages of 6 years, 10 years, and 7 months, and 8 years, respectively. The younger age seen in the Waters et al. study may have impacted the remodeling potential of the procedure and resulted in greater-than-expected results.

With the ROBINS-I analysis for the four included studies, three had “moderate” concerns of bias due to incomplete reporting of the data in the published manuscript, whereas for the remaining one study, there were no concerns, as seen in Fig. 3. At the same time, the use of Mallet scores has been the standard diagnostic assessment involving patients recovering from shoulder motion dysfunctions. The intraobserver reliability of the mallet system is excellent, while the interobserver reliability ranges from excellent to good. The ambiguous nature of the assessment, where hand to neck, hand to spine, and hand to mouth are graded by words such as “impossible” or “difficult,” which can allow for differing scores between observers [35].

## Conclusion

This systematic review and meta-analysis of non-randomized clinical studies provides supportive evidence that the L’Episcopo procedure, utilizing Latissimus Dorsi and Teres major muscles, may improve external rotation and abduction in patients with brachial plexus birth injury (BPBI). While the smaller sample size limits the strength of the conclusion, the findings support its potential role in long-term functional recovery. Future well-designed clinical studies comparing L’Episcopo and other variations using mallet scores and range of motion values are needed to provide conclusive evidence of electing a gold standard surgical approach in patients with brachial plexus birth injuries.

## Data Availability

Data is provided within the manuscript or supplementary information can be made available upon request to the corresponding author.

## References

[1] Jaufuraully S, Lakshmi Narasimhan A, Stott D, Attilakos G, Siassakos D (2023) A systematic review of brachial plexus injuries after caesarean birth: challenging delivery? BMC Pregnancy Childbirth 23(1):361 Published 2023 May 17. 10.1186/s12884-023-05696-1 37198580 10.1186/s12884-023-05696-1 PMC10190031

[2] O’Shea G, Patel SS, Mailey BA (2025) Brachial plexus birth injury: treatment and Interventions. Plast Surg (Oakv). Published Online January 12. 10.1177/22925503241301719 10.1177/22925503241301719 PMC11726498 39811497

[3] Nath RK, Halthore V, Somasundaram C (2014) Successful outcome of triangle Tilt as revision surgery in a pediatric obstetric brachial plexus patient with multiple previous operations. Case Rep Surg 2014:715389. 10.1155/2014/715389 25506033 10.1155/2014/715389 PMC4258343

[4] Louden E, Marcotte M, Mehlman C, Lippert W, Huang B, Paulson A (2018) Risk Factors for Brachial Plexus Birth Injury. Children (Basel). ;5(4):46. Published 2018 Mar 29. 10.3390/children5040046 10.3390/children5040046 PMC5920392 29596309

[5] Thatte MR, Mehta R (2011) Obstetric brachial plexus injury. Indian J Plast Surg 44(3):380–389. 10.4103/0970-0358.90805 22279269 10.4103/0970-0358.90805 PMC3263264

[6] Wells ME, Tihista MC, Diamond S (2022) Brachial plexus birth injury: trends in early surgical intervention over the last three decades. Plast Reconstr Surg Glob Open 10(5):e4346 Published 2022 May 23. 10.1097/GOX.0000000000004346 35620493 10.1097/GOX.0000000000004346 PMC9126517

[7] MacIsaac MF, Wright JM, Le NK et al (2025) Surgical treatment of neonatal brachial plexus palsy: A cohort study using the pediatric health information system (PHIS) database. Childs Nerv Syst 41:45. https://doi-org.ezproxy.rowan10.1007/s00381-024-06709-w 10.1007/s00381-024-06709-w 39666031

[8] Pondaag W, Malessy MJ (2006) Recovery of hand function following nerve grafting and transfer in obstetric brachial plexus lesions. J Neurosurg 105(1 Suppl):33–40. 10.3171/ped.2006.105.1.33 16871868 10.3171/ped.2006.105.1.33

[9] Gilbert A, Pivato G, Kheiralla T (2006) Long-term results of primary repair of brachial plexus lesions in children. Microsurgery 26(4):334–342. 10.1002/micr.20248 16634084 10.1002/micr.20248

[10] Bauer AS, Kalish LA, Adamczyk MJ et al (2020) Microsurgery for brachial plexus injury before versus after 6 months of age: results of the multicenter treatment and outcomes of brachial plexus injury (TOBI) study. J Bone Joint Surg Am 102(3):194–204. 10.2106/JBJS.18.01312 31770293 10.2106/JBJS.18.01312

[11] Brogan DM, Leversedge FJ (2019) Surgical technique and anatomical considerations for the modified l’episcopo tendon transfer. Hand (N Y) 14(1):34–41. 10.1177/1558944718803746 30295084 10.1177/1558944718803746 PMC6346371

[12] L’Episcopo JB (1934) Tendon transplantation in obstetrical paralysis. Am J Surg 25(1):122–125

[13] Hoffer MM, Wickenden R, Roper B (1978) Brachial plexus birth palsies. Results of tendon transfers to the rotator cuff. J Bone Joint Surg Am 60(5):691–695 681392

[14] Jain NS, Barr ML, Kim D, Jones NF (2024) Tendon transfers, nerve Grafts, and nerve transfers for isolated radial nerve palsy: A systematic review and analysis. Hand (N Y) 19(3):343–351. 10.1177/15589447221150516 36692098 10.1177/15589447221150516 PMC11067830

[15] Page MJ, McKenzie JE, Bossuyt PM et al (2021) The PRISMA 2020 statement: an updated guideline for reporting systematic reviews. BMJ 372:n71 Published 2021 Mar 29. 10.1136/bmj.n71 33782057 10.1136/bmj.n71 PMC8005924

[16] Bae DS, Waters PM, Zurakowski D (2003) Reliability of three classification systems measuring active motion in brachial plexus birth palsy. J Bone Joint Surg 85(9):1733–1738 12954832 10.2106/00004623-200309000-00012

[17] Sullivan GM, Feinn R (2012) Using effect Size-or why the P value is not enough. J Grad Med Educ 4(3):279–282. 10.4300/JGME-D-12-00156.1 23997866 10.4300/JGME-D-12-00156.1 PMC3444174

[18] Richardson M, Garner P, Donegan S (2019) Interpretation of subgroup analyses in systematic reviews: A tutorial. Clin Epidemiol Global Health 7(2):192–198. 10.1016/j.cegh.2018.05.005

[19] Topley MT, Russo SA, Chafetz RS, Zlotolow DA, Kozin SH, Richards JG (2022) Scapulothoracic and glenohumeral contributions to humerothoracic kinematics in single versus double tendon transfers in patients with brachial plexus birth injury. J Hand Surg Am 47(9):897. 10.1016/j.jhsa.2021.06.026 10.1016/j.jhsa.2021.06.026 34489135

[20] De Luna Cabrai JR, Crepaldi BE, de Sambuy MT, da Costa AC, Abdouni YA, Chakkour I (2015) Evaluation of upper-limb function in patients with obstetric palsy after modified Sever-L’episcopo procedure. Rev Bras Ortop 47(4):451–454 published 2015 dec 8. 10.1016/s2255-4971(15)30127-0 27047849 10.1016/S2255-4971(15)30127-0 PMC4799431

[21] Waters PM, Bae DS (2005) Effect of tendon transfers and extra-articular soft-tissue balancing on glenohumeral development in brachial plexus birth palsy. J Bone Joint Surg Am 87(2):320–325. 10.2106/JBJS.C.01614 15687154 10.2106/JBJS.C.01614

[22] Berköz HÖ, Kozanoğlu E, Aydın A, Özkan S, Akalın BE, Solakoglu S (2023) The effect of botulinum toxin application on latissimus dorsi and Teres major muscles in patients with brachial plexus birth palsy: an electron microscopic and clinical study. Doğumsal Brakiyal Pleksus felcinde botulinum Toksini uygulamasının latissimus dorsi ve Teres majör kasları üzerindeki etkisi: elektron Mikroskopik ve klinik çalışma. Ulus Travma Acil Cerrahi Derg 29(4):493–498. 10.14744/tjtes.2022.19406 36995203 10.14744/tjtes.2022.19406 PMC10214890

[23] Prasad M (2024) Introduction to the GRADE tool for rating certainty in evidence and recommendations. Clin Epidemiol Global Health 25:101484. 10.1016/j.cegh.2023.101484

[24] Higgins JP, Thomas J, Chandler J, Cumpston M, Li T, Page MJ, Welch VA (eds) (2024) (s). Cochrane Handbook for Systematic Reviews of Interventions Version 6.5 (updated August 2024). Cochrane, Available from training.cochrane.org/handbook 10.1002/14651858.ED000142 PMC10284251 31643080

[25] McGuinness LA, Higgins JPT (2020) Risk-of-bias visualization (robvis): an R package and Shiny web app for visualizing risk-of-bias assessments. Res Syn Meth 1–7. 10.1002/jrsm.1411 10.1002/jrsm.1411 32336025

[26] Mehlman CT, DeVoe WB, Lippert WC, Michaud LJ, Allgier AJ, Foad SL (2011) Arthroscopically assisted Sever-L’Episcopo procedure improves clinical and radiographic outcomes in neonatal brachial plexus palsy patients. J Pediatr Orthop 31(3):341–351. 10.1097/BPO.0b013e31820cada8 21415698 10.1097/BPO.0b013e31820cada8

[27] Immerman IMD, Valencia, Herbert RN, DiTaranto CFA†, DelSole PMD†, BS* EM, Glait SMD, Price AE, MD*,†; Grossman JAI, MD FACS*,†. Subscapularis Slide Correction of the Shoulder Internal Rotation Contracture After Brachial Plexus Birth Injury: Technique and Outcomes. Techniques in Hand & Upper Extremity Surgery 17(1):p 52–56, March 2013. | 10.1097/BTH.0b013e31827b4a23 10.1097/BTH.0b013e31827b4a23 23423238

[28] El-Gammal TA, Saleh WR, El-Sayed A, Kotb MM, Imam HM, Fathi NA (2006) Tendon transfer around the shoulder in obstetric brachial plexus paralysis: clinical and computed tomographic study. J Pediatr Orthop 26(5):641–646. 10.1097/01.bpo.0000229975.86188.c4 16932105 10.1097/01.bpo.0000229975.86188.c4

[29] De Joode S, Germawi L, Schotanus M et al (2021) Improved long-term functional outcome after a latissimus dorsi transfer with or without subscapularis muscle lengthening or release. Acta Orthop Belg 87(1):151–157 34129769

[30] Ibrahim MR, Abdelmaksoud IM, Ahmad MH, Semaya AE (2023) Comparing the results of latissimus dorsi versus Teres major transfer in children with obstetric brachial plexus injury and residual shoulder sequelae. Ann Plast Surg 90(2):144–150. 10.1097/SAP.0000000000003434 36688857 10.1097/SAP.0000000000003434

[31] Oztürk K, Bülbül M, Demir BB, Büyükkurt CD, Ayanoğlu S, Esenyel CZ (2010) Reconstruction of shoulder abduction and external rotation with latissimus dorsi and Teres major transfer in obstetric brachial plexus palsy. Acta Orthop Traumatol Turc 44(3):186–193. 10.3944/AOTT.2010.2332 21088458 10.3944/AOTT.2010.2332

[32] Lahiji FA, Tahririan MA, Karami M, Madadi F, Emami M, Maleki A (2017) Transfer of pectoralis major to subscapularis in the management of brachial plexus birth palsy sequels. J Pediatr Orthop 37(5):305–310. 10.1097/BPO.0000000000000648 26368856 10.1097/BPO.0000000000000648

[33] Kirby DJ, Buchalter DB, Santiesteban L et al (2024) Long-Term results of isolated latissimus dorsi to rotator cuff transfer in brachial plexus birth injury. J Brachial Plex Peripher Nerve Inj 19(1):e13–e19 Published 2024 Jun 12. 10.1055/s-0044-1786817 38868463 10.1055/s-0044-1786817 PMC11168807

[34] Werthel JD, Moraiti C, Leclère FM, Oberlin C, Elkholti K, Corcia T (2018) Long-term results of latissimus dorsi transfer for internal rotation contracture of the shoulder in patients with obstetric brachial plexus injury. JSES Open Access 2(3):159–164. 10.1016/j.jses.2018.05.002 30675588 10.1016/j.jses.2018.05.002 PMC6334879

[35] van der Sluijs JA, van Doorn-Loogman MH, Ritt MJ, Wuisman PI (2006) Interobserver reliability of the mallet score. J Pediatr Orthop B 15(5):324–327 16891958 10.1097/01202412-200609000-00004

